# Cerebrovascular responses to submaximal exercise in women with COPD

**DOI:** 10.1186/1471-2466-14-99

**Published:** 2014-06-05

**Authors:** Sara E Hartmann, Richard Leigh, Marc J Poulin

**Affiliations:** 1 Department of Physiology and Pharmacology, Faculty of Medicine, University of Calgary, Calgary, AB, Canada; 2 Hotchkiss Brain Institute, Faculty of Medicine, University of Calgary, Calgary, AB, Canada; 3 Department of Medicine, University of Calgary, Calgary, AB, Canada; 4 Snyder Institute of Infection, Immunity, and Inflammation, University of Calgary, Calgary, AB, Canada; 5 Faculty of Kinesiology, University of Calgary, Calgary, AB, Canada; 6 Department of Clinical Neurosciences, Faculty of Medicine, University of Calgary, Calgary, AB, Canada; 7 Libin Cardiovascular Institute of Alberta, Faculty of Medicine, University of Calgary, Calgary, AB, Canada

**Keywords:** COPD, Exercise, Transcranial Doppler ultrasound, Cerebral blood flow, Women

## Abstract

**Background:**

COPD patients have decreased physical fitness, and have an increased risk of vascular disease. In the general population, fitness is positively associated with resting cerebral blood flow velocity, however, little is known about the cerebrovascular response during exercise particularly in COPD patients. We hypothesized that COPD patients would have lower cerebral blood flow during exercise secondary to decreased physical fitness and underlying vascular disease.

**Methods:**

Cardiopulmonary exercise testing was conducted in 11 women with GOLD stage I-II COPD, and 11 healthy controls to assess fitness. Cerebro- and cardio-vascular responses were compared between groups during two steady-state exercise tests (50% peak O_2_ consumption and 30 W). The main outcome variable was peak middle cerebral artery blood flow velocity ($\bar{V}P$) during exercise using transcranial Doppler ultrasonography.

**Results:**

Physical fitness was decreased in COPD patients. $\bar{V}P$ was comparable between COPD and controls (25 ± 22% *versus* 15 ± 13%, respectively; P > 0.05) when exercising at the same relative intensity, despite patients having higher blood pressure and greater arterial desaturation. However, $\bar{V}P$ was elevated in COPD (31 ± 26% *versus* 13 ± 10%; *P* ≤ 0.05) when exercising at the same workload as controls.

**Conclusions:**

Our results are contradictory to our *a-priori* hypothesis, suggesting that during matched intensity exercise, cerebral blood flow velocity is similar between COPD and controls. However, exercise at a modestly greater workload imposes a large physical demand to COPD patients, resulting in increased CBF compared to controls. Normal activities of daily living may therefore impose a large cerebrovascular demand in COPD patients, consequently reducing their cerebrovascular reserve capacity.

## Background

The regulation of cerebral blood flow (CBF) is imperative for adequate delivery of O_2_ and energy supply to the brain. During cerebral activation and increased metabolism, cerebral arterioles dilate, thereby increasing CBF, and ultimately O_2_ delivery. This process of neurovascular coupling (i.e., neural activation coupled with an adequate increase in blood flow) is challenged during dynamic exercise. Factors such as age and physical fitness have been suggested to play an important role in this regulatory process both at rest [1], and during exercise [2].

The reduction of physical activity levels and exercise capacity in COPD patients [3-5] may pose a risk to patients’ cerebrovascular health. Even in the early stages of COPD, patients with mild and moderate obstruction are reported to have reduced exercise capacity [6]. Evidence suggests that patients with moderate COPD are already at an increased risk of cardiovascular disease and stroke, thereby suggesting an underlying vascular pathology. We have previously reported decreased CBF responsiveness to CO_2_ in women with COPD, suggesting reduced cerebral dilatory function [7].

Cycle ergometry testing offers a functional and translational tool to test integrated responses during exercise. COPD patients may exhibit arterial hypercapnia [8] and hypoxemia, exaggerated vasopressor response [9], and ventilatory limitations [10] during exercise – which all have implications on the regulation of CBF. Furthermore, greater distribution of cardiac output to respiratory or peripheral muscles could impact the blood flow delivery to the brain. Presently, few studies have focused on the cerebrovascular response of COPD patients during exercise [11,12], and of these studies, focus has been on exercise limitations. It is currently unknown if the cerebrovascular response to exercise in COPD patients differs from that of a healthy control group.

We therefore tested the hypothesis that women with mild-moderate COPD would have reduced cerebral blood flow during exercise, secondary to reduced physical fitness. Women were of particular interest in this study due to a shift in the disease burden from men to women [13], and comparatively, women report worse symptoms for a similar severity of disease to men, and for any given age, have lower aerobic capacity. Additionally, we chose to focus on patients with mild-moderate COPD because this demographic represents approximately half of all cases of COPD [14].

## Methods

### Study subjects

Twenty-three postmenopausal women (COPD = 11, Healthy Control = 12) between the ages of 55–79 years old were recruited for participation in this study. Control subjects were recruited from the community, and COPD patients were recruited from participating outpatient medical clinics within the Calgary Health Region. All study participants visited the Laboratory of Human Cerebrovascular Physiology at the University of Calgary (1103 m elevation) for two testing sessions. Subjects were instructed to refrain from eating or drinking 4 hours prior to each testing session. All study participants provided written, informed consent. Ethical approval was obtained by the Conjoint Health Research Ethics Board at the University of Calgary (Ethics ID: E-22138).

Since there are currently no published studies describing the CBF response in COPD during exercise, compared to healthy controls, we based our sample size using data from our previous study [7] describing changes in $\bar{V}P$ during hypercapnia. We expected a similar difference in $\bar{V}P$ between groups (Control = 37% vs. COPD = 19%). Therefore, a sample size of n = 10 in each group was needed to detect a significant difference with a power of 95%, and a two-tailed α of 0.05. It is likely that there are similar physiological mechanisms involved in the regulation of blood flow during exercise and hypercapnia, and reason that this power calculation would be an appropriate estimation of sample size.

#### Inclusion criteria

Patients were eligible for inclusion into the COPD cohort if they had physician-diagnosed GOLD stage I-II COPD, a smoking history >10 pack-years and, chronic airflow obstruction (FEV_1_/FVC <70%; FEV_1_ ≥ 50% predicted). To control for the immediate and lasting cardiovascular effects of nicotine, patients needed to be ex-smokers (>1 year). Additional criteria included: post-menopausal for ≥ 12 months, able to walk independently outside or on stairs, and a BMI < 35 kg m^-2^. Study subjects were allowed to be on long- or short-acting bronchodilators, either alone, or in combination with inhaled corticosteroids. Exclusion criteria included heart/chest pain upon physical exertion, surgery or trauma within previous 6 months, history of myocardial infarction, angina, arrhythmia, valve disease, chronic heart failure, history of stroke, cardiovascular or cerebrovascular disease, history of chronic headaches/migraines, history of blood clots/thrombosis, and patients who were on domiciliary oxygen.

Participating control subjects were sedentary (< 3 planned exercise sessions/week), post-menopausal, and had no history of lung disease, and no significant history of smoking (< 1 pack-year lifetime smoking history). Additional exclusion criteria conformed to that as outlined above for COPD patients.

### Study design

Subjects visited the laboratory on two occasions, and followed the same study protocol and procedures. On the first visit, volunteers completed a medical screening questionnaire, pulmonary function test, and cardiopulmonary exercise test (CPET) to assess physical fitness. Volunteers returned to the laboratory to complete the main experimental session involving CBF measurements during exercise. The main exercise protocol consisted of two bouts of 6-minutes each of exercise on a modified semi-supine cycle ergometer (~70 degrees upright) (Exercise 1 [EX1 = 50% ${\dot{V}O}_{2\;\mathrm{peak}}$ and Exercise 2 [EX2 = 30 Watts]), as previously described [15,16]. The workload at 50% ${\dot{V}O}_{2\;\mathrm{peak}}$ was intended to observe the peak CBF response, while an absolute value of 30 Watts was selected to represent the intensity of common activities of daily living, comparable to household work and light gardening (~3 metabolic equivalents; METS). The exercise periods were separated by a 6-minute rest/recovery period to allow physiological variables to return to baseline (BL).

#### Pulmonary function testing

Spirometry, measures of lung volumes and single-breath diffusion capacity were completed according to ATS guidelines [17-19].

#### Cardiopulmonary exercise test

Peak oxygen uptake (${\dot{V}O}_{2\;\mathrm{peak}}$) was assessed by a CPET performed on a modified semi-reclined cycle ergometer, according to ATS guidelines [20]. The test was initiated with a 3-minute warm-up period of unloaded cycling, immediately followed by an increase in workload to 20 W, with progressive increments of either 10 or 15 W·min^-1^ (COPD patients or controls, respectively). Ratings of perceived dyspnea and leg fatigue were scored using a modified Borg Scale every 2 minutes by the participant. Breath-by-breath metabolic and respiratory variables were collected using dedicated software (BreatheM v2.38, Oxford) and measured by mass spectrometry (AMIS 2000; Innovision, Odense, Denmark). Continuous measures of heart rate via 3-lead ECG (Micromon 7142 monitor; Kontron Medical, Milton Keynes, UK), and arterial oxygen saturation (Sao_2_) (Model 3900; Datex-Ohmeda, Louisville, CO) were recorded.

#### Protocol to measure the cerebral blood flow response to submaximal exercise

Middle cerebral artery (MCA) blood flow velocity was continuously measured using a 2-MHz pulsed Doppler Ultrasound system (TC22, SciMed, Bristol, England) as previously described [16]. Peak middle cerebral artery velocity ($\bar{V}P$) was used as a surrogate for CBF. Heart rate was collected using a 3-lead ECG (Micromon 7142B monitor; Kontron Keynes, UK), and continuous beat-beat blood pressure was measured using finger pulse photoplethysmography (Portapress; TPD Biomedical Instrumentation, Amsterdam, The Netherlands). Arterial oxygen saturation was collected using finger pulse oximetry (Model 3900; Datex-Ohmeda, Louisville, CO).

#### Respiratory measures

Subjects breathed through a mouthpiece with their nose occluded. Respiratory gas concentrations were sampled at the mouth at 100Hz using mass spectrometry (AMIS 2000; Innovision, Odense, Denmark). Respiratory volumes were measured with a turbine and volume transducer (VMM-400; Interface Associates, Laguna Niguel, CA), and respiratory flow direction and timing were obtained with a pneumotachograph (RSS100-HR, Hans Rudolf, Kansas City, MO, USA).

### Data analysis

#### Analysis

Physiological data were collected on a beat-beat and breath-breath basis using dedicated software (BreatheM v2.38, University Laboratory of Physiology, Oxford, UK) and later averaged into 15-second bins. Baseline data were averaged over 2-minutes immediately preceding exercise. To achieve steady state parameters, the last 60-seconds of exercise (i.e., minute 5 to 6) were used in the stage averages. The main outcome variable in this study was $\bar{V}P$. Secondary outcomes were ${\dot{V}O}_{2\;\mathrm{peak}}$, mean blood pressure (MBP), and cerebrovascular conductance ($\mathrm{CVC}=\bar{V}P/\mathrm{MBP}$).

#### Statistical procedures

Using statistical software (SPSS Version 20.0, SPSS Inc., Chicago, IL, USA), mean between-group differences in subject characteristics, pulmonary function data, and endpoints of the maximal cardiopulmonary exercise test were determined using independent t-tests. Paired t-tests were used to assess baseline (BL) differences within groups (i.e., COPD BL1 vs. COPD BL2). A 2×2 mixed factorial repeated measures analysis of variance was used to assess the main effect of independent variables “*condition”* (i.e., BL1 vs. EX1 or BL2 vs. EX2), and “*grouping*” (i.e., COPD or CONTROL), and the interaction between *condition* x *grouping* on physiological outcomes (i.e., $\bar{V}P$, CVC, MBP, HR, Sao_2_).

Relationships were assessed exclusively within groups using Pearson’s correlation coefficient to investigate fitness status (${\dot{V}O}_{2\;\mathrm{peak}}$), disease severity (FEV_1pred_, D_LCO_), Sao_2_, Petco_2_, and MBP on cerebrovascular variables at rest and during exercise. Data are presented as mean ± SD, with significance set at α-level ≤ 0.05. Two tailed tests were performed in all analyses.

## Results

Two COPD patients were not able to complete the EX2 protocol; however, their data from EX1 was included in the study analysis. One control participant was excluded due to lack of a suitable CBF signal. We therefore compared the exercise responses between 11 COPD and 11 control participants in EX1, and 9 COPD and 11 control participants in EX2.

### Baseline characteristics

Physical characteristics and results of pulmonary function tests are listed in Table 1. Groups were well matched for age, weight and BMI (P > 0.05). By study design, COPD patients had a significantly greater smoking history than controls (*P* < 0.01). Based on post-spirometric FEV_1_ predicted values, disease severity ranged from GOLD I-II (mild to moderate) (51% to 86%) in COPD patients (n = 9, GOLD II). COPD patients had reduced diffusion capacity (D_LCO_), and increased hyperinflation (FRC, IC/TLC) compared to control subjects (*P* < 0.01), who all had normal lung function*.*

**Table 1:** Subject characteristics

	**COPD (n = 11)**		**Controls (n = 11)**	
***Physical Characteristics***	Mean		Mean	
Age, years	69.6 ± 5.6		64.2 ± 7.1	
Weight, kg	68.8 ± 12.8		69.4 ± 9.6	
BMI, kg·m^-2^	26.8 ± 4.6		25.6 ± 2.7	
Smoking Pack-years	38.4 ± 12.9 †		0.2 ± 0.3	
***Lung Function***		% Predicted		% Predicted
FEV_1_, L	1.38 ± 0.24 †	67	2.50 ± 0.34	107
FVC, L	2.64 ± 0.51 *	97	3.24 ± 0.38	108
TLC, L	5.53 ± 1.04	115	5.12 ± 0.45	101
RV, L	2.97 ± 0.78 †	146	1.81 ± 0.30	88
FRC, L	3.40 ± 0.77 †	124	2.48 ± 0.34	86
IC, L	2.07 ± 0.50 *		2.52 ± 0.43	
IC/TLC,%	37 †		49	
RV/TLC,%	53 †		35	
D_LCO_, ml · min^-1^ · mmHg^-1^	14.2 ± 3.3 †	72	21.1 ± 3.1	107

*Abbreviations*: BMI, Body mass index; FEV_1_, Forced vital capacity in 1 second; FVC, Forced vital capacity; TLC, Total lung capacity; RV, Residual volume; FRC, Functional residual capacity; IC, Inspiratory capacity; D_LCO_, Diffusion lung capacity. Significantly different from Controls at *P* ≤ 0.05 (*) and *P* ≤ 0.01 (†).

### Cardiopulmonary exercise test

Results of the cardiopulmonary exercise test are summarized in Table 2. Mean ${\dot{V}O}_{2\;\mathrm{peak}}$ and peak power output was lower in patients with COPD. Arterial oxygen saturation was significantly decreased in COPD patients with exercise, but not in controls (*P* < 0.01). At end of exercise, COPD patients achieved similar predicted heart rate values to controls, suggesting a ventilatory limitation to exercise, rather than any cardiovascular impairment (*P* > 0.05)*.*

**Table 2:** Summary of Cardiopulmonary Exercise Test Endpoints in COPD and controls

	**COPD (n = 11)**	**Controls (n = 11)**
${\dot{V}O}_{2\;\mathrm{peak}}$		
L·min^-1^	1.10 ± 0.34 *	1.52 ± 0.41
mL·min^-1^·kg^-1^	16.1 ± 4.6 *	22.6 ± 7.0
$\dot{V}O_{2\;\mathrm{peak}\;\mathrm{pred}},\%$	85 ± 29	106 ± 28
$\dot{V}{\mathrm{CO}}_{2\;\mathrm{peak}},L\cdot \mathrm{mi}n^{‐1}$	1.08 ± 0.37 †	1.64 ± 0.50
${\dot{V}}_{E\;\mathrm{peak}},L\cdot \mathrm{mi}n^{‐1}$	40.7 ± 9.5 *	62.5 ± 21.1
${{\dot{V}}_{E}}_{\mathrm{peak}}/\mathrm{MVV},\%$	84 ± 16	71 ± 19
B*f*, breaths·min^-1^	31 ± 5	35 ± 8
Sao_2_,%	89.5 ± 4.3 †	94.5 ± 2.0
Heart rate		
HR _peak_, beats·min^-1^	121 ± 14 *	140 ± 20
HR _age pred_,%	81 ± 10	90 ± 13
O_2_ Pulse (mlbeat^-1^)	9.1 ± 2.7	10.8 ± 2.0
Workrate		
WR _peak_, Watts	57 ± 13 †	117 ± 34
WR _pred_,%	63 ± 21 †	103 ± 20
Borg dyspnea (0–10)	5.2 ± 1.1	5.8 ± 1.8
Borg leg Fatigue (0–10)	4.3 ± 1.2	5.6 ± 1.6

*Abbreviations*: ${\dot{V}O}_{2\;\mathrm{peak}}$, Peak volume of O_2_ consumed; ${\dot{V}O}_{2\;\mathrm{peak}}$, Peak volume of CO_2_ produced; ${\dot{V}}_{E}$_peak_, Peak expired ventilation rate; B*f*_peak_, Peak breathing frequency; Sao_2_, Arterial oxygen saturation; HR _peak_, Peak heart rate; O_2_ Pulse: Oxygen pulse (${\dot{V}O}_{2}$/HR); WR _peak_, Peak work rate. Significantly different from controls at *P* ≤ 0.05 (*) and *P* ≤ 0.01 (†).

### Responses to exercise at a relative workload: 50% $\dot{V}O_{2\;\mathrm{peak}}$ (EX1)

The target intensity for EX1 was set at 50% ${\dot{V}O}_{2\;\mathrm{peak}}$ for 6 minutes (EX1). Accordingly, groups exercised at a similar exercise intensity (${\dot{V}O}_{2\;\mathrm{peak}}$: 69% (COPD) *versus* 50% (controls); *P* > 0.05). Metabolic cost was not different between groups in EX1 (${\dot{V}O}_{2}$: 0.69 L⋅min^-1^ (COPD) *versus* 0.74 L⋅min^-1^ (controls); *P* > 0.05). Despite similar workrates between groups (COPD: 15 W *versus* Controls: 23 W; *P* > 0.05), COPD patients exercised at a greater capacity of their WR_peak_ (COPD: 25% *versus* Controls: 18%; *P* ≤ 0.05). Patients and controls had similar Petco_2_ and Peto_2_ at rest, and in response to EX1 (*P* > 0.05).

The cerebro-and cardiovascular responses to EX1 are summarized in Table 3. There were no differences between resting cerebro- or cardio-vascular variables between groups (*P* > 0.05). The two-factor analysis of variance revealed that there was a significant main effect of exercise in increasing $\bar{V}P$, HR, and MBP (*P* ≤ 0.05). CVC was not affected by exercise (*P* > 0.05). There was a significant group x exercise interaction for MBP (mmHg) (*P* ≤ 0.05), but not $\bar{V}P$ (cm·s^-1^) (*P =* 0.17). Sao_2_ decreased with exercise, and this effect was the greatest in COPD patients, as exhibited by an exercise x group interaction (*P* ≤ 0.05).

**Table 3:** Workrate, metabolic and respiratory parameters during rest and submaximal exercise

	**Exercise 1 (Relative Workload)**				**Exercise 2 (Absolute Workload)**			
	**COPD (n=11)**		**Control (n=11)**		**COPD (n=9)**		**Control (n=11)**	
	**BL1**	**EX1**	**BL1**	**EX1**	**BL2**	**EX2**	**B** **L** **2**	**EX2**
**Workrate, W**	-	15 ± 20	-	23 ± 19	-	30	-	30
**WR**_**max**_**,%**	-	25 ± 37 †	-	18 ± 14	-	48 ± 7 †	-	25 ± 11
*Metabolic and Respiratory*								
${\dot{V}}_{E},L\cdot \mathrm{mi}n^{-1}$	8.9 ± 2.3	22.4 ± 7.1	7.3 ± 1.4	20.5 ± 5.1	11.8 ± 2.8*	27.9 ± 5.8	9.0 ± 1.4	21.0 ± 6.6
$\dot{V}O_{2},L\cdot \mathrm{mi}n^{-1}$	0.28 ± 0.12	0.69 ± 0.28	0.27 ± 0.05	0.74 ± 0.17	0.27 ± 0.04	0.78 ± 0.22	0.28 ± 0.05	0.74 ± 0.19
$\dot{V}O_{2\mathrm{peak}},\%$	28 ± 10*	69 ± 31	19 ± 6	50 ± 11	25 ± 9	74 ± 30	20 ± 7	52 ± 20
$\dot{V}CO_{2},L\cdot \mathrm{mi}n^{-1}$	0.23 ± 0.09	0.58 ± 0.24	0.22 ± 0.04	0.63 ± 0.17	0.26 ± 0.05	0.69 ± 0.18	0.25 ± 0.04	0.63 ± 0.17
**P** **etco**_**2**_**, mmHg**	32.5 ± 3.2	34.9 ± 4.1	33.8 ± 2.1	36.0 ± 3.0	31.1 ± 3.3	34.4 ± 3.7	33.1 ± 1.9	35.4 ± 2.7
**P** **eto**_**2**_**, mmHg**	89.3 ± 5.0	87.6 ± 4.9	88.2 ± 4.8	86.3 ± 5.1	95.2 ± 5.2	89.4 ± 5.5	92.5 ± 5.2	88.0 ± 5.8

*Abbreviations*: WR_max_, Work rate maximum; ${\dot{V}}_{E}$, Minute ventilation; ${\dot{V}O}_{2}$, Volume of oxygen uptake; ${\dot{V}\mathrm{CO}}_{2}$, Volume of carbon dioxide production; Petco_2_, Pressure of end-tidal CO_2_; Peto_2_, Pressure of end-tidal O_2._ *P ≤ 0.05 (different from controls at baseline). † *P* ≤ 0.05 (different between groups).

In summary, exercise increased $\bar{V}P$ similarly in COPD (42.8 to 50.2 cm·s^-1^) and controls (44.2 to 50.2 cm·s^-1^), while working at a similar exercise intensity (*P* > 0.05), despite COPD patients exhibiting a greater exercise-pressor response and greater arterial oxygen desaturation.

### Responses to exercise at absolute workload: 30 watts (EX2)

Physiological description of the exercise protocol is summarized in Table 4. All subjects cycled at a workrate of 30 W for 6 minutes (EX2). By design, there was no difference between ${\dot{V}O}_{2}$ between groups (${\dot{V}O}_{2}$: 0.78 L⋅min^-1^ (COPD) *versus* 0.74 L⋅min^-1^ (controls); *P* > 0.05). There was a trend for COPD patients to be exercising at a greater relative exercise intensity than controls (${\dot{V}O}_{2\;\mathrm{peak}}$: 74% (COPD) *versus* 52% (controls); *P* = 0.08). Despite the same workrate between groups, COPD patients exercised at a greater capacity of their WR_peak_ (COPD: 48% *versus* Controls: 25%; *P* ≤ 0.01). Patients and controls had similar Petco_2_ and Peto_2_ at rest, and in response to EX2.

**Table 4:** Cerebro- and cardio-vascular responses to submaximal exercise in COPD and controls

	**Exercise 1**					**Exercise 2**				
	**COPD**		**Control**			**COPD**		**Control**		
	**BL1**	**EX1**	**BL1**	**EX1**	***P***	**BL2**	**EX2**	**BL2**	**EX2**	***P***
*Cerebrovascular*										
$\bar{V}P,\mathrm{cm}\cdot s^{-1}$	42.8 ± 10.4	52.4 ± 10.3	44.2 ± 13.6	50.2 ± 13.3	*	40.2 ± 12.1	51.1 ± 11.5	43.8 ± 12.5	48.7 ± 11.9	* †
**%**		25 ± 22		15 ± 13	*		31 ± 26		13 ± 10	* # †
**CVC, cm·s**^**-1**^**·mmHg**^**-1**^	0.46 ± 0.11	0.47 ± 0.09	0.49 ± 0.15	0.50 ± 0.15		0.45 ± 0.13	0.46 ± 0.90	0.47 ± 0.14	0.49 ± 0.15	
**%**		5 ± 15		3 ± 8			9 ± 27		4 ± 8	
*Cardiovascular*										
**HR, bpm**	67 ± 7	93 ± 11	66 ± 8	90 ± 14	*	69 ± 8	101 ± 10	71 ± 12	92 ± 15	* †
**MBP, mmHg**	94.5 ± 8.7	113.7 ± 18.1	92.0 ± 13.2	101.8 ± 12.7	* †	95.4 ± 9.6	112.9 ± 27.0	94.5 ± 15.2	103.7 ± 24.2	*
**%**		20 ± 13		11 ± 8	*		18 ± 22		9 ± 10	*
**Sa** **o**_**2**_**,%**	94 ± 2	91 ± 4	95 ± 1	94 ± 2	* # †	95 ± 1	91 ± 4	96 ± 1	95 ± 1	* # †

*Abbreviations*: $\bar{V}P$; Peak cerebral blood flow velocity; CVC, cerebrovascular conductance (CVC = $\bar{V}P$/MBP); MBP, Mean arterial blood pressure; HR, Heart rate; Sao_2_, Arterial oxygen saturation; * *P* ≤ 0.05 (main effect of e*xercise*); # *P* ≤ 0.05 (main effect of g*rouping*); † *P* ≤ 0.05 (e*xercise* x g*rouping* interaction).

**Figure 1:**
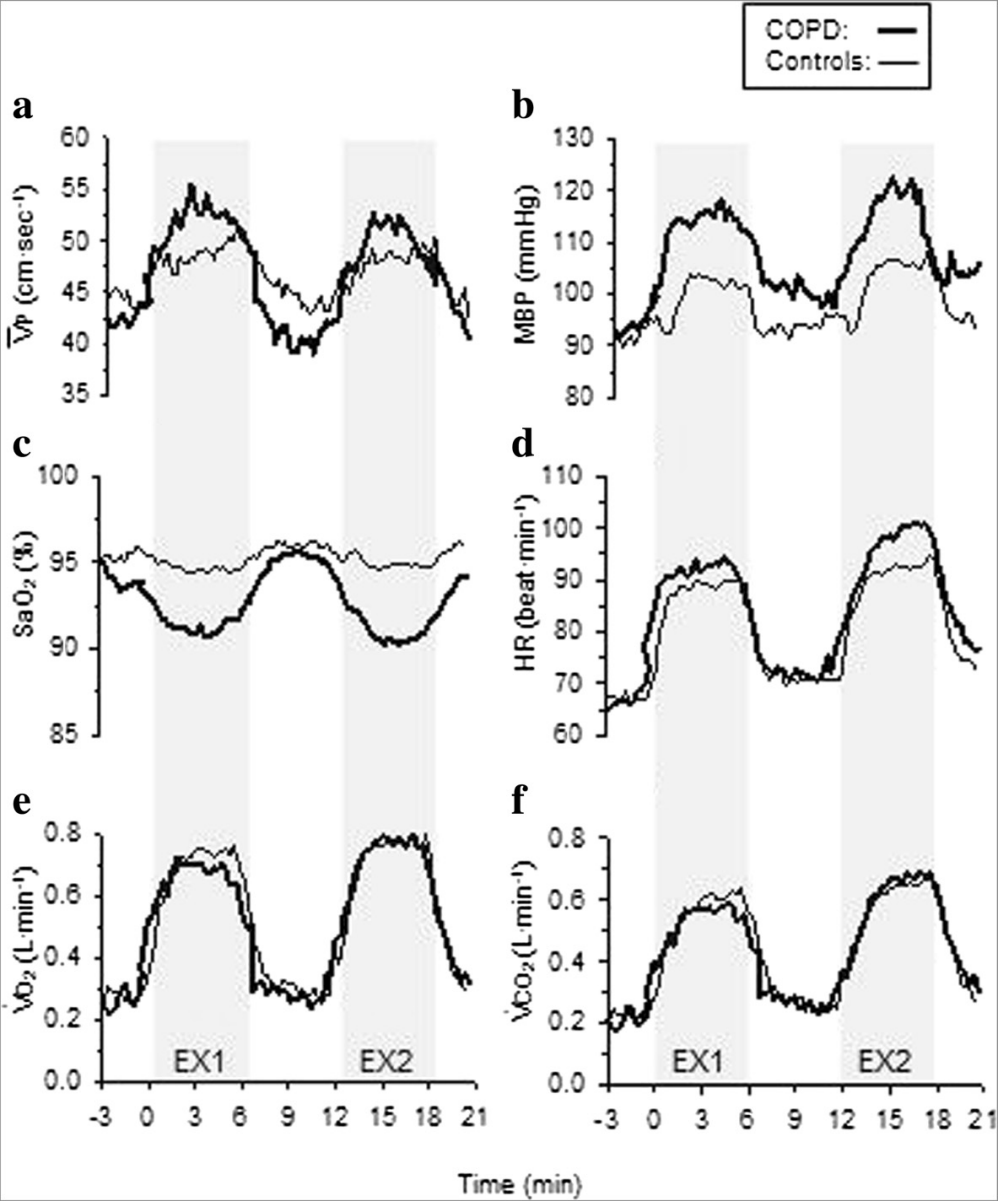
**Continuous representation of the physiological responses to two, 6-minute exercise periods in women with moderate COPD (thick line) and healthy age-matched controls (thin line).** Exercise 1 (EX1; 50% ${\dot{V}O}_{2\;\mathrm{peak}}$) and Exercise 2 (EX2; 30 Watts) were separated by 6-minutes of resting recovery. **a)** Peak cerebral blood flow velocity in the middle cerebral artery ($\bar{V}P$); **b)** mean arterial blood pressure (MBP); **c)** arterial oxygen saturation (Sao_2_); **d)** heart rate (HR); **e)** volume of oxygen consumption (${\dot{V}O}_{2}$); and **f)** volume of carbon dioxide production (${\dot{V}\mathrm{CO}}_{2}$). Data represent 15-second averages. Cycling exercise periods are shaded.

The cerebro-and cardiovascular responses to EX2 are summarized in Table 3, and individual stage means are presented in Figure 1. There were no differences between resting cerebro- or cardio-vascular variables between groups (*P* > 0.05). The two-factor analysis of variance revealed that there was a significant main effect of exercise in increasing $\bar{V}P$, HR, and MBP, and decreasing Sao_2_ (*P* ≤ 0.05). There was a significant group by exercise interaction for $\bar{V}P$ (cm·s^-1^) (*P* ≤ 0.05). Exercise significantly decreased Sao_2_ overall (*P* < 0.01), but had the greatest effect in COPD patients whom desaturated at a greater rate than controls as exhibited statistically by a grouping by exercise interaction effect (*P* <0.01).

In summary, exercise increased $\bar{V}P$ to a greater extent in COPD patients (40.2 to 51.1 cm·s^-1^) compared to controls (43.8 to 48.7 cm·s^-1^), while working at 30 Watts (and an equivalent ${\dot{V}O}_{2}$). At this workload, however, there was a trend for COPD patients to be working at a higher percentage of maximum capacity than the control subjects. Blood pressure increased with exercise, but changes were similar between groups.

### Relationships between fitness, disease severity, and cerebrovascular measurements during exercise

At rest, there was no relationship between fitness status, disease severity, Sao_2_, Petco_2_, and blood pressure on cerebrovascular measures. In both groups, and in both exercise conditions, participants with lower baseline $\bar{V}P$ were more likely to have low exercise $\bar{V}P$, and *vice versa* (EX1: *R*^*2*^ = 0.75, *P* < 0.01; EX2: *R*^*2*^ = 0.78, *P* <0.01). Furthermore, individuals with lower baseline $\bar{V}P$ were more likely to have a greater absolute increase in exercise $\bar{V}P$ (EX1: *R*^*2*^ = 0.18, *P* ≤ 0.05; EX2: *R*^*2*^ = 0.29, *P* < 0.01). Percent change in MBP was positively associated with change in $\bar{V}P$ (EX1: *R*^*2*^ = 0.36, *P* < 0.01; EX2: *R*^*2*^ = 0.22, *P* ≤ 0.05). There was no relationship between cerebrovascular variables and Sao_2_ during exercise. In EX2, the change in Petco_2_ with exercise was positively related to the changes in $\bar{V}P$ and CVC ($\bar{V}P$: *R*^*2*^ = 0.37, *P* < 0.01; CVC: *R*^*2*^ = 0.40, respectively, *P* < 0.01). There was no significant correlations between ${\dot{V}O}_{2\;\mathrm{peak}}$ and $\bar{V}P$, MBP, or CVC (*P* > 0.05) in either EX1 or EX2 (all subjects combined).

## Discussion

Our main findings are that 1) when patients with mild to moderate COPD exercise at the same intensity to healthy controls, the CBF response is similar; and 2) when comparing CBF at a matched O_2_ demand (i.e., 30 Watts), CBF is elevated in this cohort of COPD patients. From these results, it is apparent that COPD does not have a detrimental effect on cerebral blood flow during moderate intensity exercise. The mechanisms underlying the increased CBF in COPD at higher workloads needs to be investigated further.

Cerebral blood flow velocity has consistently been shown to increase in healthy individuals during mild to moderate exercise intensity [16,21-23], and seems to be intensity dependent [24]. Although mechanisms are not fully elucidated [25], important regulators of the exercise-induced CBF response include: cortical activation [26], arterial blood pressure [27], cardiac output [21], and blood gases (particularly $Pa_{CO_{{}_{2}}}$) [28]. Comparative studies are particularly lacking in the area of ageing and disease. Consequential alterations of CBF are apparent with age, however, physical fitness has been shown to offset these changes [1,15]. Aerobic capacity appears to be an important determinant in cerebrovascular health with ageing, as it has been shown to be positively correlated with cerebrovascular CO_2_ reactivity [29], and better cognitive outcomes [15]. Current opinion suggests that COPD represents a case of advanced ageing [30], which may make these patients particularly susceptible to alterations in cerebrovascular responses.

We reasoned that the known vascular consequences associated with COPD, including endothelial dysfunction [31], and increased arterial stiffness [32] would play a detrimental role in the CBF response to exercise. A recent study suggests that physical fitness (i.e., 6MWD) has a favorable effect on endothelial function in COPD patients [33]. We have furthermore shown that women with moderate COPD exhibit a nearly two-fold reduction in the expected CBF response to CO_2_[7]. It is likely that during a more modest cerebrovascular challenge (e.g., submaximal exercise *versus* hypercapnia), the cerebrovascular response remains intact, despite underlying vascular pathologies that may exist.

We found patients to have decreases in Sao_2_ during exercise in response to maximal and submaximal exercise. As hypoxia is known to stimulate blood flow, it is interesting that we did not find a relationship between individuals with lower Sao_2_ during exercise and increased CBF. However, it is possible that increased CBF, as seen in COPD during more intense exercise (e.g., EX2), could be partially driven by decreased Sao_2_, in a regulatory effort to increase cerebral oxygen delivery.

Results from a study investigating cerebral hemodynamics in severe COPD during exhaustive exercise suggest increased frontal cortex O_2_ delivery at the end of exhaustive normoxic exercise, in conjunction with increased CBF, despite exercise induced hypoxemia [11]. These results would suggest a regulatory difference, as it has alternatively been shown in healthy hypoxemic individuals that CBF is decreased (from rest) during exhaustive exercise [34]. The differences between these studies was explained by the lack of hyperventilation in COPD which maintained (and even increased) $Pa_{CO_{{}_{2}}}$, causing a vasodilatory response. However, caution is warranted as this study does not allow for an accurate comparison with an age-matched control group. The “normal” hyperventilation-induced hypocapnia evident during high-intensity exercise as been shown to be blunted in healthy older individuals, which in turn leads to maintenance of $Pa_{CO_{{}_{2}}}$, and CBF [35]. In patients with terminal lung disease, Jensen et al. [12] has shown continuous increases in CBF, with a slight reduction in cerebral oxygenation at end-exercise in room air, without changes in $Pa_{CO_{{}_{2}}}$. It is therefore likely, that the CBF response in COPD depends on several aspects, such as disease severity, blood gases, exercise intensity, and physical fitness. We are hesitant to draw conclusions based on $Pa_{CO_{{}_{2}}}$, as this variable was not measured in our study. However, COPD patients continue to increase ${\dot{V}}_{E}$ in EX2, which would not support the hypoventilation/increased $Pa_{CO_{{}_{2}}}$ theory.

Cardio-pulmonary interactions during exercise in COPD have the potential to have an adverse influence on CBF. Increased expiratory loading and dynamic hyperinflation (DH) during exercise may impair venous return, and consequently lead to a reduction in right heart preload, and ultimately stroke volume. O_2_ pulse is commonly used as a surrogate measure of stroke volume (and cardiac efficiency). Studies have shown DH to be negatively correlated with O_2_ Pulse during incremental exercise in COPD [36,37]. Furthermore, resting hyperinflation (IC/TLC) was associated with a reduction of O_2_ pulse at peak exercise. We did not find a difference in O_2_ pulse at peak exercise between COPD patients and controls (Table 2), and take this to indicate that cardiac function was not impaired in our patients. Despite COPD patients in this study having significantly lower IC/TLC ratio at rest (37% versus 49%), this level of hyperinflation is noticeably less when compared to results of the aforementioned studies which reported DH during exercise. Furthermore, the role of DH in mild-moderate COPD remains to be clearly defined. Lastly, although cardiac output remains an important determinant in cerebrovascular regulation, reductions in cardiac output (by lower body negative pressure) reduce CBF, but seem to be less important during exercise compared to the resting state, which has been explained as a counter-regulatory activation of the sympathetic nervous system [38]. Overall, we speculate, that DH is not a major contributor to the determinants of CBF in our sample population. As this remains beyond the scope of this study, it should be considered in future studies.

One strength of our study is that it includes data comparing the physiological response during both as exercise challenge that is a fixed percentage of each person’s maximal workload, and an absolute workload - which represents a value that may be a more realistic occurrence in activities of daily living. Although we found that patients with mild and moderate COPD have a normal CBF response at 50% ${\dot{V}O}_{2\;\mathrm{peak}}$, it is important to highlight that this workload is equivalent to ~2.9 METS, which would equate to light housework such as self care, washing dishes, and walking to a parking lot [39]. When physical demands are slightly increased to 30 Watts (~3.2 METS), these COPD patients further increase CBF. The importance of absolute requirements are highlighted by Paterson *et al*. [40] who report that a relative ${\dot{V}O}_{2}$ of ~15 mL O_2_·kg^-1^·min^-1^ is required for independent living in the aging population. During moderate activities (e.g., vigorous cleaning, sweeping, etc.), COPD patients would be exposed to increased aerobic demand, which may have cerebrovascular implications, including a reduced cerebrovascular reserve capacity compared to healthy controls. This remains highly speculative, and the implications of this are unclear at this time. Although we do not have measures of habitual activity, we have previously shown a significant, positive agreement between self-reported physical and ${\dot{V}O}_{2\max}$ in healthy, older women [41]. In this same study, fitness was correlated with better vascular outcomes during exercise [15]. We therefore suspect that individuals in the present study who have a higher ${\dot{V}O}_{2\max}$ are also more physically active, although this needs to be confirmed in future research. Conversely, it is possible that frequent increases in CBF may pose a vascular benefit to COPD patients, whereby increases in CBF result in shear stress on the vascular endothelium, thus providing beneficial adaptations through the up-regulation of nitric oxide synthase [42]. In contrast, modest activities increase CBF in COPD and may limit the supply of blood flow available for additional challenges, such as cognitive tasks.

Our selection of mild – moderate COPD patients includes 9 of 11 patients with GOLD II, moderate COPD. Of these 11 patients, 2 could not complete a 6-minute workload of 30 Watts. We believe that more severe patients would have further difficulty completing 2×6-min bouts of cycling exercise. Further, our selected cohort of COPD patients represents the patients that are most likely either still physically active, or still have the potential to become more active, and we would therefore gain the greatest insight into studying this population. Women in particular have lower ${\dot{V}O}_{2\max}$, smaller lung volumes, and exhibit greater dyspnea at a similar ${\dot{V}O}_{2}$ compared to men [43]. Finally, a homogeneous sample of women reduces additional heterogeneity between subjects, and is particularly important in this smaller sample size.

### Limitations

Transcranial Doppler ultrasound is a commonly used, non-invasive tool to measure the velocity of blood in the MCA. The MCA provides ~ 80% of the blood flow to the brain, and perfuses the frontal and temporal lobe, including the motor-sensory cortex, which is activated during exercise. This technique offers several advantages over other methodologies because of its non-invasive properties, and high temporal resolution [44,45]. One drawback of using this method, however, is the assumption that the MCA diameter remains constant, allowing us to infer that velocity represents flow. The regulation of CBF is thought to occur in the small arterioles, such that the large basal cerebral arteries maintain a constant diameter thus, providing an accurate representation of CBF [16,45].

We firstly chose to compare the steady-state CBF response at a relative exercise intensity. Secondarily, we studied the physiological response between COPD and controls at an absolute workload. We acknowledge that at an absolute workload participants are working at a different relative exercise capacity, which introduces confounding variables. It is therefore difficult to interpret the specific mechanisms involved in the observed differences in CBF during this stage. However, we believe this exercise workload to be of significant importance and these findings notable.

Lastly, a limitation of our study was the absence of arterial blood gases as part of our methodology. We therefore are precluded from making a conclusion regarding the influence of $Pa_{CO_{{}_{2}}}$ on the regulation of CBF during exercise in COPD.

## Conclusion

Cerebral blood flow responsiveness appears to be normal in patients with mild to moderate COPD compared to healthy controls when exercising at a moderate intensity (50% ${\dot{V}O}_{2\max}$), but not when exercising at a slightly greater, matched workload (i.e., 30 Watts). It is likely that decreased physical fitness contribute to this. These results have implications for normal activities of daily living such as household activities in which COPD patients would likely experience greater increases in CBF, thereby decreasing the cerebral vascular reserve.

## Abbreviations

BL: baseline stage; CBF: cerebral blood flow velocity; CPET: Cardio-pulmonary exercise test; COPD: Chronic obstructive pulmonary disease; D_LCO_: Diffusion capacity of the lung for carbon monoxide; EX: Exercise stage; FEV_1_: Forced expiratory volume in 1 second; MBP: Mean blood pressure; MCA: Middle cerebral artery; METS: Metabolic equivalents; $Pa_{CO_{{}_{2}}}$: Arterial CO_2_ pressure; Petco_2_: End-tidal CO_2_ pressure; Sao_2_: Arterial oxygen saturation; $\bar{V}P$: Peak cerebral blood flow velocity; ${\dot{V}O}_{2}$: Oxygen uptake.

## Competing interests

The authors declare that they have no competing interests.

## Authors’ contributions

SEH contributed to study design, data collection, data analysis, and interpretation, and wrote the first draft of the manuscript. RL contributed to study design, medical coverage and patient referral, data interpretation, and critically reviewed the manuscript; MJP contributed to study design, fundraising, study supervision, data interpretation, and critically reviewed the manuscript. All authors read and approved the final manuscript.

## Pre-publication history

The pre-publication history for this paper can be accessed here:

http://www.biomedcentral.com/1471-2466/14/99/prepub
